# Overexpression of HSD17B4 exerts tumor suppressive function in adrenocortical carcinoma and is not associated with hormone excess

**DOI:** 10.18632/oncotarget.22827

**Published:** 2017-12-01

**Authors:** Guanxiong Ding, Shenghua Liu, Qiang Ding, Chenchen Feng

**Affiliations:** ^1^ Department of Urology, Huashan Hospital, Fudan University, Shanghai 200040, PR China; ^2^ Fudan Institute of Urology, Fudan University, Shanghai 200040, PR China

**Keywords:** adrenocortical carcinoma, HSD17B4, tumor suppressor, p53

## Abstract

**Aim:**

Adrenocortical carcinoma (ACC) is characterized with excessive hormone production. We therefore investigated expression of hormone-related genes in ACC.

**Results:**

We queried status of 14 key genes directly involved in adrenal hormone production and found HSD17B4 expression was upregulated in 39% of ACC cases on top of all queried genes. Overexpression of HSD17B4 was significantly associate with a normo-hormonal phenotype. Constitutive HSD17B4 expression was higher in ACC cell line NCI-H295R than in adrenocortical small cell carcinoma cell line SW13. NCI-H295R cells with HSD17B4-knockdown (KD) demonstrated significantly inhibited proliferation, increased apoptosis, and increased cell cycle arrest. Enrichment analysis for mRNA expression in ACC samples with or without HSD17B4 overexpression showed significant change in p53 pathway. Replenish of HSD17B4 in SW13 cells and knockdown of HSD17B4 in H295R cells confirmed alterations in MDM4, ATR, and IE24 with alterations more contrasting in H295R cells. HSD17B4-KD inhibited cell invasion, migration and anchorage independent growth of NCI-H295R cells, but not of SW13 cells.

**Materials and Methods:**

Clinical and genetic data of ACC samples were reproduced from the ACC dataset of The Cancer Genome Atlas (TCGA) database using cBioPortal. Genes participating in adrenal hormone production were queried. Association between gene status and hormone release were studied and *in vitro* assays using RNA interference were carried out.

**Conclusions:**

Overexpression of HSD17B4 exerted tumor suppressive function in adrenocortical carcinoma and was not related to hormone excess. Crosstalk between HSD17B4 and p53 warrants further investigation.

## INTRODUCTION

Adrenocortical carcinoma (ACC) is a rare endocrine malignance at a prevalence of approximately 0.7 to 2 per million people [1, 2]. While the disease is surgically resectable at earlier stages, it is substantially aggressive at advanced stages. Multidisciplinary modalities including chemotherapy, radiotherapy, surgical intervention and adrenolytic therapy are the mainstay of treatments for patients with stage III and IV diseases, which above all confer a poor prognosis [3–5].

The cortex of adrenal gland secretes a variety of vital hormones which are produced in excessive amount in ACC and causes severe symptoms. Starting from cholesterol, the adrenal cortex produces cortisol, mineralocortisol, and sex hormones in general under meticulous mediation from the central-peripheral axis via series of enzymes. In the malignant setting, dysregulated hormone excess could lead to disrupted balance in metabolism, electrolyte equilibrium and sexual appearance. Cortisol excess, the predominant type of hormone dysregulation in ACC, may substantially compromise immune response, allow viral replication, and bring cortisol-related multi-systematic complications, all being severe health problems [6–8]. However, whether excessive hormone secretion is intrinsically pro-carcinogenic or stands solely as a byproduct facilitating ACC to a minor extent, remains unclear. The answer lies in part in the contribution of genes encoding those enzymes for hormone production as well as in cases without hormone excess, which occur in approximately 40% of cases [9].

In the current study, we aim to systematically investigate the alterations of genes encoding hormone related genes in adrenal gland and their associations with clinical hormone phenotypes, as well as their genetic relevance to other oncogenic pathways in a relatively large cohort (over 90 cases) for this rare disease, thanks to The Cancer Genome Atlas (TCGA) project, which contains comprehensive and multilevel genetic information [10]. We expect to reveal roles of such genes other than hormonal regulation and provide novel insights on the potential to develop targeted strategy in ACC.

## RESULTS

### HSD17B4 is overexpressed in ACC

**Figure 1:**
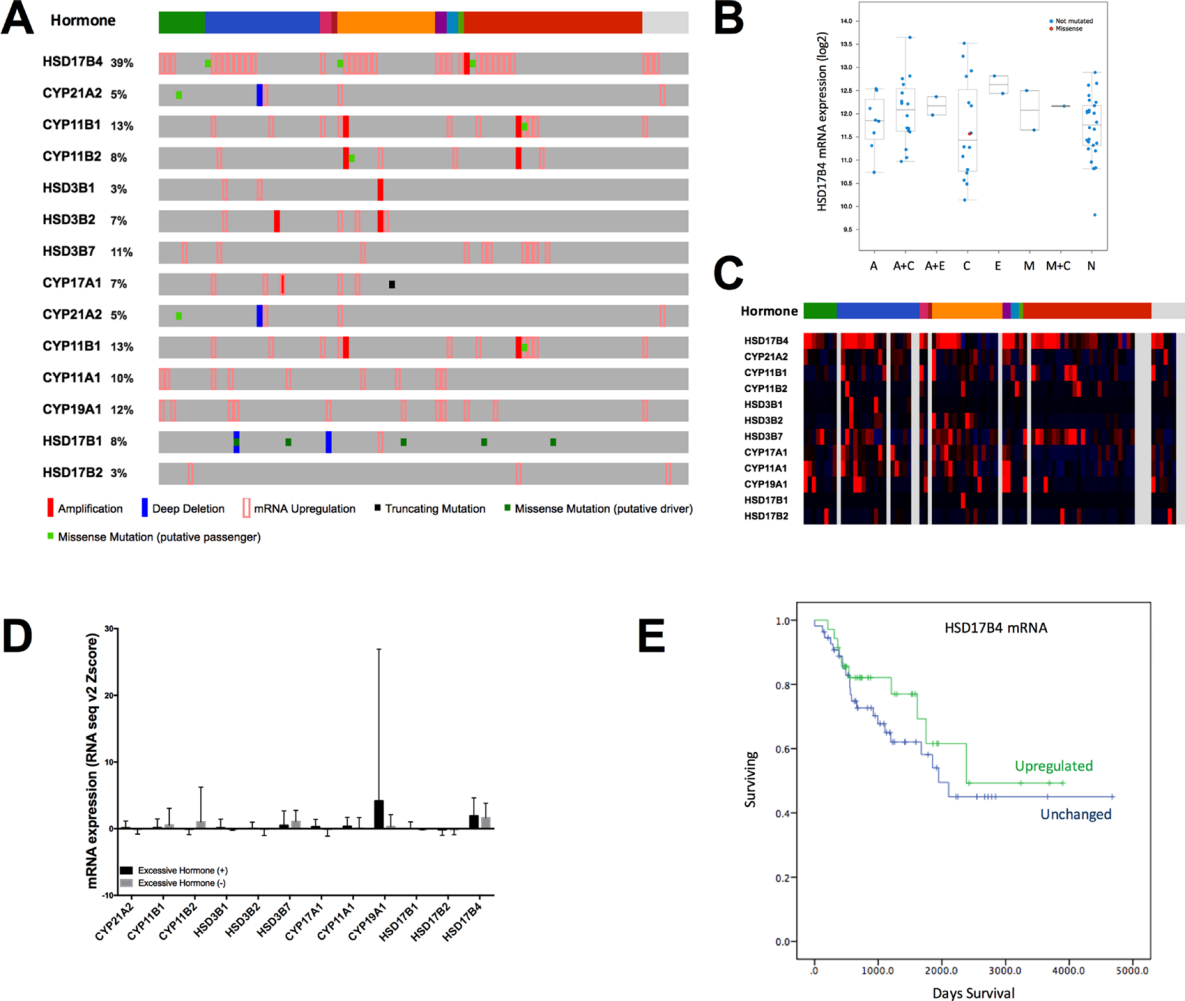
HSD17B4 is over expressed in adrenocortical carcinoma (ACC) Reproduction of TCGA ACC dataset showing (**A**) genetic alteration of a panel of adrenocortical hormone-related genes; (**B**) expression levels of a panel of adrenocortical hormone-related genes; (**C**) expression of HSD17B4 and its association with excessive hormonal type; (**D**) expression level of each gene grouped by the presence of excessive hormone; and (**E**) difference in overall survival between patients with or without HSD17B4 overexpression.

There were 14 genes encoding enzymes that participate in adrenal hormone production included in the current study, namely, CYP21A2, CYP11B1, CYP11B2, HSD3B1 HSD3B2, HSD3B7, CYP17A1, CYP21A2, CYP11B1, CYP11A1, CYP19A1, HSD17B1, HSD17B2, and HSD17B4. In general, alteration in at least one of the genes occurred in 66% of cases (61out of 92). Changes at expression level was predominant compared with mutation and CNV (Figure 1A). Of note, HSD17B4 was altered in ∼39% of cases, ranking top of all genes with overexpression in 29 cases, mutation in 3 cases and gene amplification in 1 case, respectively. We then evaluated the association between mRNA expression and hormone type and found that enhanced function of the genes was mainstay of alteration (Figure 1A–1B). Over half of the patients had excessive hormone release and cortisol was the major type (Figure 1B). Overexpression of HSD17B4 was not associated with excessive hormone release (*P* = 0.668, Figure 1B). In further analysis with breakdown of hormone types, association between HSD17B4 expression level and a certain or combination of hormones were not noticed either (*P* > 0.05 for all comparisons, Figure 1C). We then studied expression of each of the 14 genes and the association with hormone production and found that when regarded as a categorical variable, overexpression of HSD3B7 and HSD17B4 were associated with normo-hormonal phenotype while overexpression of CYP11A1 was associated with hormone excess (Table 1). However, there was no significant association detected when expression was analyzed as continuous variable (Figure 1D). In the univariate analysis for association between HSD17B4 expression and clinicopathological parameters, we found lack of significant association for adjuvant mitotane therapy (*P* = 0.092), tumor stage (*P* = 0.148), metastasis (*P* = 0.661), Weiss score (*P* = 0.143), response to primary therapy (*P* = 0.183), residual tumor status (*P* = 0.236), lymph node involvement (*P* = 0.578) or patient gender (*P* = 0.489). Tumors without necrosis showed lower HSD17B4 expression and the difference was close to statistical significance (*P* = 0.059). Patients with HSD17B4 overexpression had on average ∼50 days longer overall survival than patients without. However, the difference did not reach statistical significance (*P* = 0.699, Figure 1E).

**Table 1:** A Chi-square analysis of overexpression of hormone producing genes and presence of excessive hormone, reproduced from TCGA ACC dataset

–	–	Excessive Hormone		*P* value
–	–	-	+	
CYP21A2	-	31	49	0.1170
	+	0	4	
CYP11B1	-	26	47	0.5285
	+	5	6	
CYP11B2	-	29	48	0.6332
	+	2	5	
HSD3B1	-	31	50	0.1773
	+	0	3	
HSD3B2	-	31	47	0.0519
	+	0	6	
HSD3B7	-	24	50	**0.0208**
	+	7	3	
CYP17A1	-	31	47	0.0519
	+	0	6	
CYP21A2	-	31	49	0.1170
	+	0	4	
CYP11B1	-	26	47	0.5285
	+	5	6	
CYP11A1	-	31	44	**0.0152**
	+	0	9	
CYP19A1	-	29	45	0.2379
	+	2	8	
HSD17B1	-	29	48	0.6332
	+	2	5	
HSD17B2	-	30	52	0.6977
	+	1	1	
HSD17B4	-	9	29	**0.0225**
	+	22	24	

### HSD17B4 knockdown promotes ACC growth

**Figure 2:**
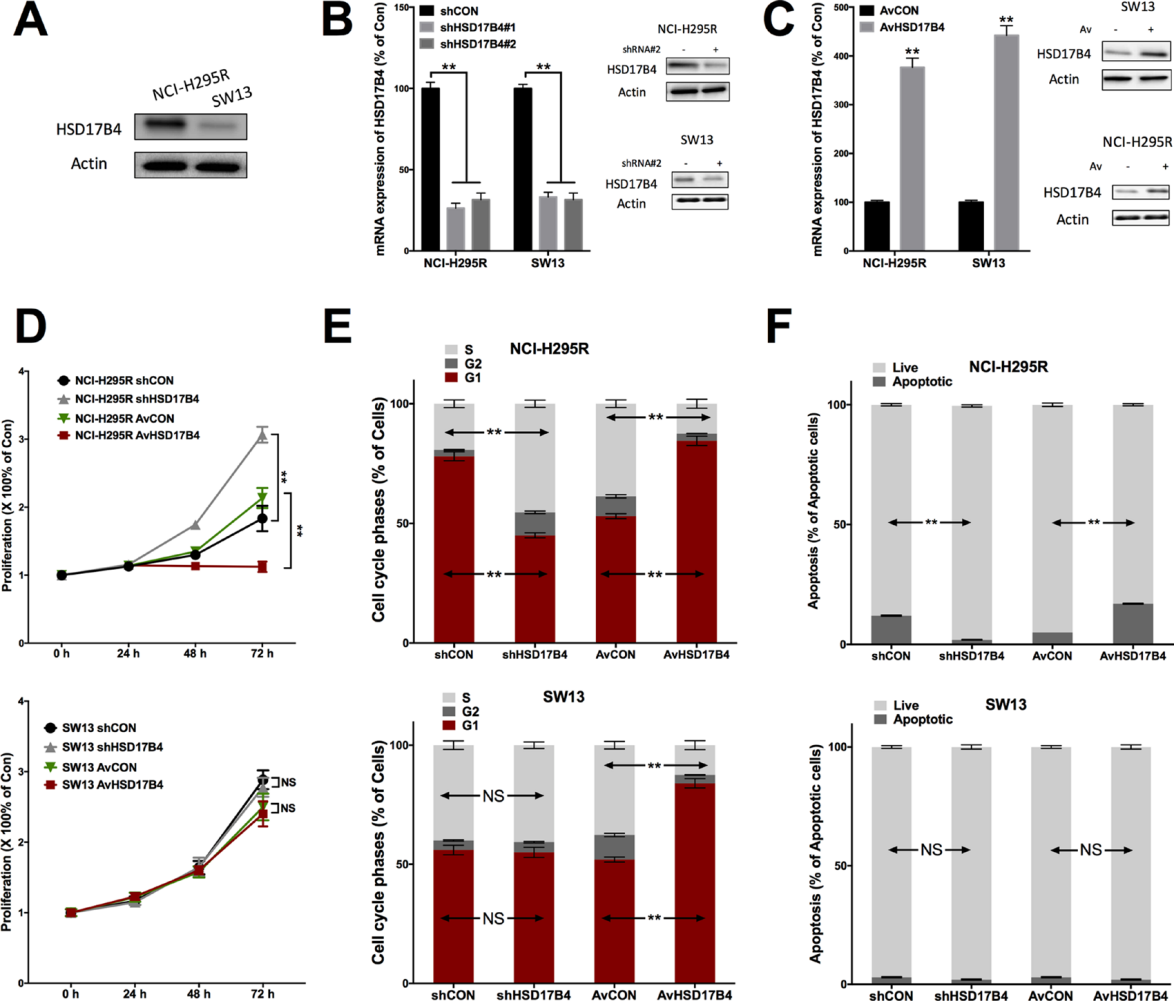
HSD17B4 exerts tumor suppressive effect in ACC cells NCI-H295R and SW13 showing (**A**) constitutive HSD17B4 level; (**B**) robust knockdown (KD) of HSD17B4 using shRNAs in both cell lines and (**C**) robust upregulation of HSD17B4 in both cell lines; HSD17B4-KD and overexpression in NCI-H295 and SW13 cells effecting on (**D**) proliferation, (**E**) cell cycle and (**F**) cell apoptosis, respectively (^**^P < 0.01, *n* = 3).

Constitutive level of HSD17B4 was studied in 2 cell lines, in which NCI-H295R is ACC cells and SW13 is small cell carcinoma of adrenal cortex. NCI-H295R cells showed substantially higher HSD27B4 level (Figure 2A). Genetic knockdown (KD) using shRNA efficiently downregulated HSD27B4 expression in both cells (Figure 2B). Adenovirus-mediated overexpression of HSD17B4 in both cells also showed high efficiency (Figure 2C). HSD27B4-KD NCI-H295R cells showed significantly increased proliferation, whilst the effect was not significant in SW13 cells (Figure 2D). Overexpression and KD of HSD17B4 induced significant increase and decrease in G1 population, respectively (Figure 2E). However, only HSD17B4 overexpression induced increased G1 population in SW13 cells and KD did not, indicating an inherent difference between the cancer types (Figure 2E). HSD17B4-KD also resulted in significant inhibition of cell apoptosis in NCI-H295R cells but not in SW13 cells (Figure 2F).

### p53 signaling is enriched in HSD17B4 upregulated cases

**Figure 3:**
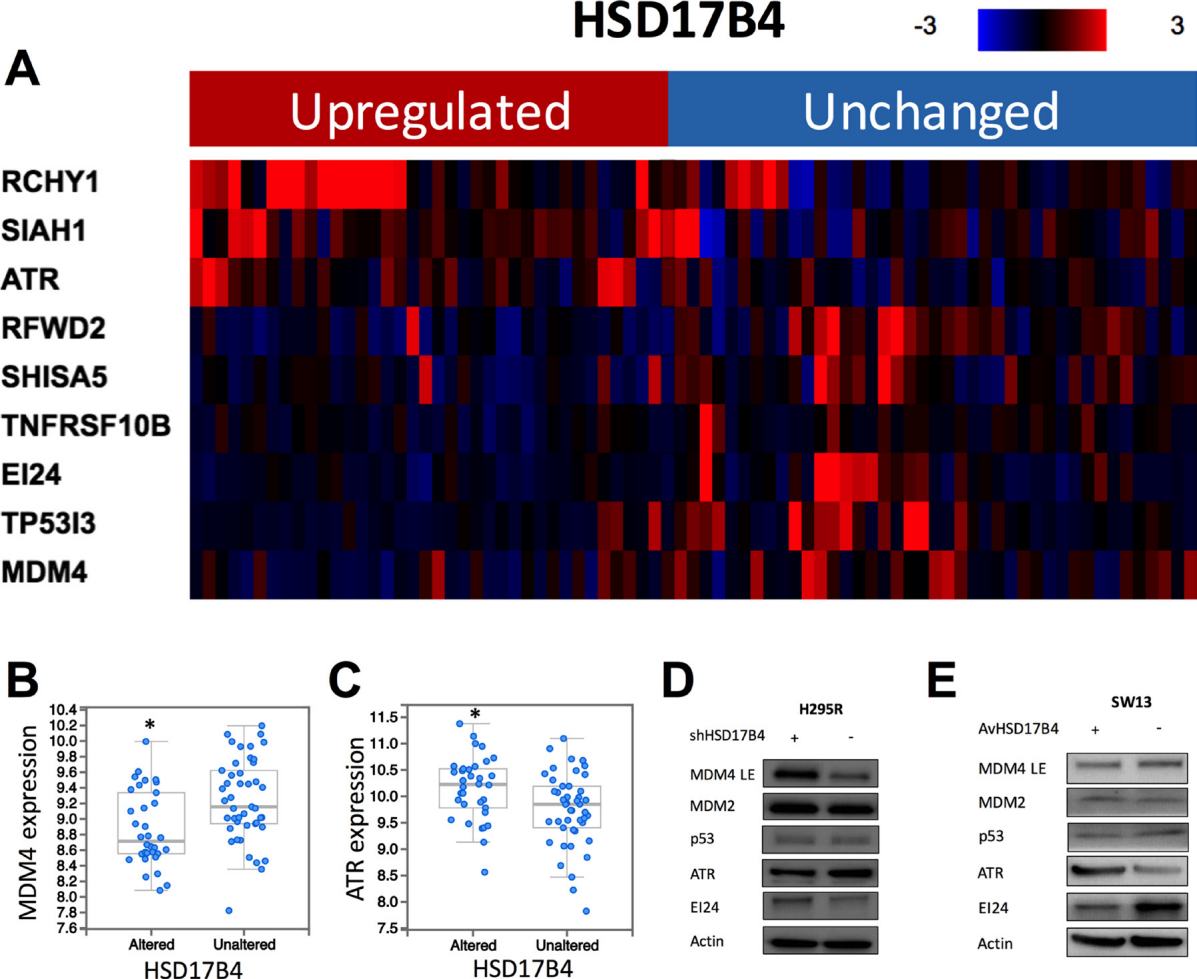
HSD17B4 overexpression is associated with p53 signaling alteration (**A**) reproduction of TCGA ACC dataset showing heatmap of p53 signaling related genes in cases with and without HSD17B4 overexpression; (**B**) MDM4 expression is significantly lower in cases with HSD17B4 overexpression; (**C**) ATR expression is significantly higher in cases with HSD17B4 overexpression; enriched genes were confirmed for protein level using western blotting in (**D**) H295R cells with and without HSD17B4-KD, and in (**E**) SW13 cells with and without HSD17B4 replenish (^*^P < 0.05).

The enrichment analysis yielded 1923 genes with expressions significantly enriched in cases with HSD17B4 overexpression. Further functional annotation revealed 77 significantly altered pathways annotated by KEGG PATHWAY database. Nine genes within p53 signaling showed significant enrichment in expression levels, including higher expressions of RCHY1, SIAH1, ATR and lower expressions of RFWD2, SHISA5, TNFRSF10B, EI24, TP53I3, and MDM4, respectively in HSD17B4 overexpressed cases (Figure 3A–3C). In validation study at translation level, we found that HSD17B4 KD in NCI-H295R cells showed higher MDM4, IE24 and lower ATR levels (Figure 3D). In SW13 cells, HSD17B4 overexpression showed the similar alteration in MDM4, IE24, and ATR levels (Figure 3E).

### HSD17B4 is associated in part with metastatic potential and tumorigenesis

**Figure 4:**
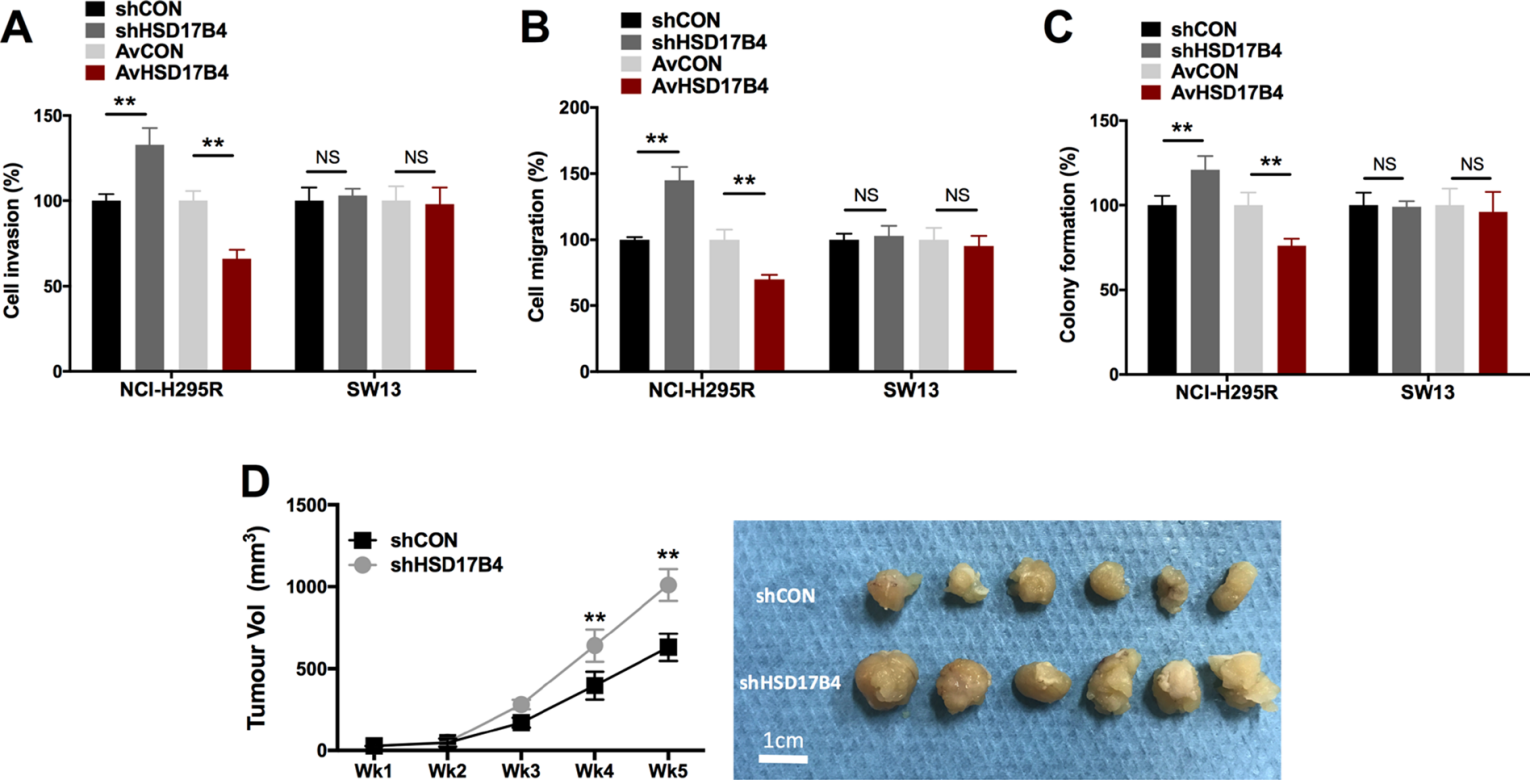
HSD17B4 altered cell motility in H295R but not in SW13 cells HSD17B4-KD and overexpression in NCI-H295 and SW13 cells effecting on (**A**) cell invasion; (**B**) cell migration; and (**C**) colony formation, respectively; (**D**) HSD17B4-KD induced increased *in vivo* tumor growth of NCI-H295R cells (^**^P < 0.01, NS = not significant, *n* = 3).

Both epithelial-mesenchymal transition (EMT) and anchorage-independent growth were indicators for metastatic potential. We found that HSD17B4-KD NCI-H295R cells showed significantly increased cell invasion and migration (Figure 4A–4B). Also, HSD17B4-KD NCI-H295R cells demonstrated significantly reduced colony formation (Figure 4C). Nonetheless, SW13 cells did not show any significant alteration in cell invasion, migration, or colony formation regardless of overexpression or KD (Figure 4A–4C). In the *in vivo* study, we observed significantly increased tumor growth in NCI-H295R cell with HSD17B4 KD (Figure 4D).

## DISCUSSION

In the current study, we found that hormone related gene HSD17B4 exerted tumor suppressive function and was not associated with hormonal phenotype of ACC. To date, our study was the first to study a relatively complete panel of adrenocortical hormone related genes in ACC and the role of HSD17B4 therein was not previously reported. While it was not surprising that overexpression was the predominant genetic alteration of those genes given that ∼60% of ACC patients suffer excessive hormonal symptoms, it was astonishing the most frequently altered gene was not associated with any excessive hormone type. The heatmap however showed an obvious association between overexpression of certain genes (i.e. HSD3B2, CYP17A1, CYP11A1, and CYP19A1) and excessive hormone. In the further statistical analysis using a categorical stratification, in which overexpression of a certain gene was strictly defined, we find that HSD17B4 was significantly associated with normo-hormonal phenotype, indicating that HSD17B4 overexpression in ACC could be responsive to combat excessive hormone production and appears quite effective. Given that excessive hormone is immunosuppressive and the effect of HSD17B4 can be, in a way, tumor suppressive by modulating hormone level. This also corresponds with the previous reports that key genes for steroidogenesis are CYP subtypes and there could be functional redundancy for the rest of genes [11–14]. Excess of hormone could bring hirsutism in female, gynecomastia in male, or Cushing syndrome that are substantially detrimental [15]. However, there lacks a clear association between excessive hormone with worsened or better prognosis [16]. It is thus possible that hormonal excess is a byproduct of ACC due to the inherent function of the adrenal gland parallel to tumorigenesis. This notion was further supported by the indication for mitotane, which serves as an antihormonal agent rather than antitumoral, implying separate regulatory pathways therein [17].

Overexpression of HSD17B4 is ubiquitous and in peripheral organs it plays a theoretical role in converting estradiol to estrone and D^5^-androstenediol to dehydroepiandrogen (DHEA), and both of which are supposed increase estrone. Given that adrenal glands provide precursors for target organ to produce terminal hormones, the lack of association between HSD17B4 and hormone in ACC can be attributed to tropism. However, carcinogenic role of HSD17B4 has been reported in other non-endocrine malignancies. Overexpression of HSD17B4 was noted in prostate cancer with significant association with worsened prognosis via possibly steroid metabolism and fatty acid oxidation [18]. In hepatocellular carcinoma, HSD17B4 was shown to act as an adaptor between inflammation and cancer cell proliferation [19]. In our study, however, HSD17B4 appears to play a tumor-suppressive role in ACC, and the effect was even cancer type restricted as the results we received using NCI-H295R cells could be only in part repeated in SW13 cells, which is not de facto ACC. Frequently altered genes towards amplified gene function (i.e. overexpression, focal amplification, etc.) do not necessary indicate pro-tumorigenic role. This could result from selection-induced subclonal heterogeneity, and/or from treatment related response. Both notions can be perceived in our findings. First, high prevalence of overexpression yet lack of association with critical clinicopathological parameters (i.e. tumor stage, survival, etc.) indicates the existence of heterogeneity. Closer association with non-necrotic phenotype indicates such genotype towards better subtype of tumor. On the other hand, a near-significant association with adjuvant mitotane use is indicative for drug-related genetic response. In further *in vitro* studies, where micro- and macro- environments were much simpler, the tumor suppressive role of HSD17B4 was amplified.

In search of the potential mechanism thereof, we analyzed expressional correlation and enrichment for HSD17B4. Surprisingly, we captured substantial long list of significantly altered pathways from the KEGG pathway. We therefore carefully screened the pathways based on the recently published studies on genomic characterization of ACC [20, 21], including the TCGA ACC project whose data were used and reproduced in our study. Although the studies provide the most solid genomic data for this rare entity so far, it remains controversial how much those significantly mutated genes (SMGs) and related pathways substantially contribute to tumorigenesis and can be actionable. Taking the previous studies into account, current evidence supports TP53, CTNNB1, and PRKAR1A related signaling playing putative “driving” roles in ACC. Our finding that key elements in p53 signaling altered in HSD17B4 overexpressed cases support the tumor suppressive role we noted. Also, our findings that genetic manipulation of HSD17B4 did not alter cell motility in SW13 cells, indicating that effect of HSD17B4 is cell- and cancer- context dependent and constitutive HSD17B4 expression is important for its function. However, the interaction between p53 signaling and HSD17B4 remains in SW13 cells. The detailed interaction warrants further investigation.

Out study entails several unanswered questions. First, regulatory mechanism and biological effect of other enzymes altered at lower frequencies remains unstudied – we are currently running studies thereon. Second, we did not investigate HSD17B4 expression in ACC cells treated with mitotane and other chemo-agents. This should be studied to evaluate the indicative role of HSD17B4 for treatment. Third, in the modern era, patient-derived cells lines can more accurately represent the current genetic spectrum than immortalized commercial cells. However, the success of establishment of patient-derived ACC cell line is opportunistic. Thus, in the current study, we used NCI-295R as the experimental model and SW13 as control. The currently available ACC cell for sale, or accessible for our group are exactly those 2 cells. Another cell line, the HAC15 cells available at ATCC (also the only ACC cell lines for sale at ATCC) is indeed a sub-clone of NCI-H295R. Last but not the least, the detailed regulatory mechanism between HSD17B4 and p53 signaling is still at large. We are now conducting mechanistic analysis on the molecular level, aiming to validate our observation.

## MATERIALS AND METHODS

### Reproduction of TCGA-ACC dataset and functional annotation

The cBioPortal online platform was used for reproduction of the TCGA-ACC database [22, 23], as previously reported [24]. We chose the dataset encompassing 92 samples and selected genomic profiles of mutations, putative copy-number alterations from GISTIC, and mRNA expression z-scores (RNA Seq V2 RSEM). We queried a series of genes that encoded key enzymes participating in adrenocortical steroid production [25, 26]. Gene alterations were visualized using OncoPrint. By adding and sorting per clinical attribute of Excess Adrenal Hormone History Type, we generated expressional heatmap of the queried genes. Expression of a certain gene categorized by hormone type was visualized by the Plot function. Also, relative expression level of each gene in cases with or without hormone excess was analyzed. We then solely queried the gene status of HSD17B4. Alteration of HSD17B4, predominantly overexpression, was analyzed for association with clinicopathological parameters (adjuvant mitotane therapy, tumor stage, metastasis, Weiss score, response to primary therapy, residual tumor status, lymph node involvement, patient gender, and overall survival). We then extracted genes enriched amongst HSD17B4-altered and –unaltered cases. Significantly altered genes (passing both *p* and *q* values) were subject to the KOBAS 3.0 system for functional annotation and pathway analysis [27, 28]. The KEGG pathway database was used.

### Cell lines and culture

The human adrenocortical cancer (NCI-H295R) and adrenocortical small cell carcinoma (SW13) cell lines were originally obtained from ATCC and cultured in DMEM medium (Thermo Scientific, Logan, UT, USA) supplemented with 20% fetal bovine serum.

### RNA interference

Both cell lines were subject to HSD17B4 knockdown (KD) using shRNAs. The sequences for HSD17B4-KD were chosen from TRC (TRCN0000220382 for shRNA#1 and TRCN0000220386 for shRNA#2). Viral transduction was performed according to standard protocol [29]. Briefly, viral particles were prepared in 293T cells. Starved cells were transduced with equal titers of enveloped particles in OptiMEM media (Thermo) supplemented with 1 μg/ml polybrene (Sigma, Deisenhofen, Germany) for 24 h. Puromycin was used for selection. Cells were also subject to HSD17B4 overexpression using adenovirus mediation. A human full-length cDNA clone of HSD17B4 on pCMV6-AC-GFP vector (Origene, Rockville, MD) was used and ligated to a secondary vector. After recombination procedure, HSD17B4-bearing and control adenovirus were generated. Cells infected were evaluated for multiplicities of infection (MOI) and cytopathy, and 100 MOI was designated as optimal.

### Quantitative RT-PCR

Expression of HSD17B4 was quantified using Realtime RT-PCR, per established protocol [30]. Primers for HSD17B4 were as follows: Forward 5′- TGA GGG ATC GTT CCT TTG CTA -3′; Reverse 5′- CGT GTC ACT TGG AAT GAA CCC -3′. We used our in-house GAPDH primers for internal reference [30]. After conversion of extracted mRNA to cDNA, real-time PCR procedure with SYBR Green Premix Ex Taq (TaKaRa) in a 20-μL system were run on ABI 7500n (Applied Biosystems, Forster City, CA). For each sample, the average value of threshold cycle was normalized to GAPDH level with the formula, 2^−△△Ct^.

### Western blotting

Standard western blotting procedure was followed [31]. Briefly, Cell lysates were separated with 12% sodium dodecyl sulfate polyacrylamide gel electrophoresis and transferred onto nitrocellulose membranes, which were subsequently blocked for 4 hrs. Membranes were then incubated with non-fat milk-diluted primary antibodies: Rabbit polyclonal anti-HSD17B4 (Sigma); mouse monoclonal antibody against MDM2 (Abcam, Cambridge, UK); Anti-MDM4 TRUEMAB antibody (Origene); anti-p53 mouse monoclonal antibody (Abcam); rabbit polyclonal antibody against ATR (Abcam); and rabbit polyclonal anti-IE24 antibody (Abcam). Corresponding secondary antibodies were applied followed by electrochemiluminescence (ECL) processing. MDM4 was subject to long exposure (LE) due to relatively weak signal.

### Proliferation assay

The crystal violet assay was used to evaluate cell proliferation. Cells were seeded in 96-well plates at a density of 2500 cells/well for 0, 24, 48 and 72 h. At each time point, medium was gently removed and cells were fixed with formalin. Cells were then stained with 0.05% CV for 30 min. Methanol was applied and plates were read at absorbance of OD 540 nm. All readings were normalized to 0 h status.

### Cell cycle and apoptosis

The cell cycle and apoptosis in the current study were measured using flow cytometry. For cell cycle analysis, cells were trypsinized and treated with cell cycle staining buffer (MultiSciences Biotech, Hangzhou, China) for 15 min. Suspension were then subject to flow cytometry on a BD FACSCanto flow cytometer. For apoptosis, cells were stained with Annexin V-fluorescein (BD Pharmingen, Pasig City, Philippines) and propidium iodide (PI) (BD) for 15 min at room temperature. Samples were then analyzed with flow cytometry to determine percentages of apoptotic cells using Annexin V/PI indication.

### Migration, invasion and colony formation

Both migration and invasion were studied with Transwell assays. Roughly 1000 cells were cultured in the Transwell inserts either coated (for invasion) or uncoated (for migration) with Matrigel. The inserts were placed in a 24-well plate filled with complete medium. Cells that penetrated to the underside surfaces of the inserts were fixed and stained with the crystal violet. The mean of cell number of three high power fields for each condition was calculated. Colony formation assay was used to profile anchorage-independent growth. Briefly, 1000 cells were seeded in the mixture of 10% FBS-containing medium and 0.4% agarose, which was layered on top of 0.6% agar in 20% FBS-containing medium. On top of the agarose was 1 ml of complete medium that was changed every 3 days. Two weeks later, the plates were stained with 0.005% of crystal violet for 1 h. Colonies were counted microscopically.

### Xenograft model

Twenty male athymic nude mice at 6 weeks of age were bred in SPF (special pathogen-free) grade laboratory. Mice were randomly divided into 2 groups (Treatment versus control). A total of 1.5 × 10^7^ NCI-H295R cancer cells with or without HAD17B4-KD, resuspended in 100 ml of PBS were injected subcutaneously at the left axilla of each mouse. Mice were monitored every 3 days for general condition and tumor growth and all were sacrificed on Day. Tumor size was calculated with the formula, Length × Width^2^ × 0.5236.

### Statistical analysis

All data were processed with the SPSS ver.21 and Prism Graphpad ver.6 software. For comparison between means of 2 cohorts, the Student’s *t*-test was used. For survival analysis, the Kaplan Meier plot and Log rank test were used. Correlation was studied using the Spearman test. Enrichment analyses were automatically computed by the cBioPortal platform and KOBAS 3.0 system. All data were presented as mean ± standard deviation (SD). All *in vitro* studies were performed in triplicates with 3 replications. The *P* value of <.05 was accepted as statistically significant and the *Q* value of <.05 was accepted for the threshold of false discovery rate (FDR).

## CONCLUSIONS

ACC is a rare but aggressive disease. It is often associated with excessive hormone production. We find adrenocortical hormone related gene HSD17B4 is overexpressed at high frequency in ACC but it is not associated with hormone excess. Overexpression of HSD17B4 exerts tumor suppressive role in ACC and it may play a role in p53 signaling.
